# TGF-β1 promotes expression of fibrosis-related genes through the induction of histone variant H3.3 and histone chaperone HIRA

**DOI:** 10.1038/s41598-018-32518-8

**Published:** 2018-09-19

**Authors:** Toshihiro Shindo, Shigehiro Doi, Ayumu Nakashima, Kensuke Sasaki, Koji Arihiro, Takao Masaki

**Affiliations:** 1 Department of Nephrology, Hiroshima University Hospital, Hiroshima, Japan; 2 Department of Pathology, Hiroshima University Hospital, Hiroshima, Japan

## Abstract

Renal fibrosis is a histological manifestation that occurs in almost every type of chronic kidney disease. Histone variant H3.3 and its chaperone, histone cell cycle regulation defective homolog A (HIRA), serve as epigenetic marks that regulate transcriptional activity. In this study, we assessed the roles of histone H3.3 and HIRA in unilateral ureteral-obstruction (UUO) mice. In UUO mice, the levels of histone H3.3 and HIRA were significantly upregulated in the kidneys. These upregulated levels were decreased by a TGF-β1 neutralizing antibody. TGF-β1 induced histone H3.3 and HIRA expression in vitro via a Smad3-dependent pathway in normal rat kidney (NRK)−52E cells. Additionally, knockdown of HIRA expression decreased histone H3.3 expression and fibrogenesis in NRK-52E cells after TGF-β1 stimulation. Chromatin immunoprecipitation analysis revealed that promoters of fibrosis-related genes were immunoprecipitated with both histone H3.3 and HIRA in NRK-52E cells. Lastly, in human kidney biopsies from patients diagnosed with IgA nephropathy, histone H3.3 and HIRA immunostaining correlated positively with areas of fibrosis and estimated glomerular filtration rate. In conclusion, TGF-β1 induces expression of histone H3.3 and HIRA, which regulates expression of fibrosis-related genes.

## Introduction

Chronic kidney disease (CKD) is estimated to occur in 13–15% of the population in developed countries^1,2^ and it exerts a substantial socioeconomic burden worldwide. Importantly, progressive CKD not only results in end-stage kidney disease (ESKD) but also increases mortality from any cause^3–9^, particularly from cardiovascular disease^6–9^. To suppress the progression of CKD, inhibitors of the renin-angiotensin-aldosterone system have been developed^10^. However, these treatments have limited effects and many patients finally succumb to irreversible ESKD and require dialysis or kidney transplantation.

Irrespective of the initial causes, renal fibrosis occurs in ESKD^11^. Pathologically, renal fibrosis is characterized by excessive proliferation of α-smooth muscle actin (α-SMA)-positive myofibroblasts and overproduction of extracellular matrix (ECM), which eventually leads to renal failure^12,13^. Among a number of cytokines, transforming growth factor (TGF)-β1 is a fundamental agent in the progression of renal fibrosis^14,15^. Recently, we demonstrated that TGF-β1-induced histone H3 lysine 4 monomethylation (H3K4me1) promotes the transcriptional activity of genes involved in fibrosis through the induction of SET domain–containing lysine methyltransferase 7/9 (SET7/9)^16^. However, SET7/9 possibly affects all histone tails of histone H3; therefore, the epigenetic mechanism by which TGF-β1 specifically regulates transcriptional activity of fibrosis-related genes is not fully understood.

In chromatin, histones exist as an octamer that contains two molecules of each core histone, H2A, H2B, H3, and H4^17^. Histone variants are incorporated by histone chaperones in place of canonical histones and function as a transcriptional landmark. The histone variant, H3.3, is specifically enriched at transcriptionally active genes^18–21^, and histone cell cycle regulation defective homolog A (HIRA) is the specific histone chaperone of histone H3.3^22–24^. Renal fibrosis is considered to be a state in which fibrosis-related genes are activated by TGF-β1, raising the possibility that TGF-β1-regulated histone variants contribute to renal fibrosis^16^. Moreover, a recent study revealed that histone H3.3 overlaps with H3K4me1 and serves as an enhancer of active genes^25^. These findings led us to the hypothesis that TGF-β1-induced HIRA plays a role in renal fibrosis through incorporation of histone H3.3.

In this study, we used ureter obstruction (UUO) model mice, a well-established rodent model of TGF-β1-mediated renal fibrosis^26^. We show that TGF-β1 induces histone H3.3 and HIRA via a Smad3-dependent pathway. We also demonstrate that HIRA is involved in the induction of histone H3.3 and in TGF-β1-induced α-SMA expression *in vitro*. Furthermore, chromatin immunoprecipitation (ChIP) analysis revealed that TGF-β1 increases the immunoprecipitation of histone H3.3 and HIRA on promoters of fibrosis-related genes. Finally, renal expression of histone H3.3 and HIRA correlated with fibrotic areas and estimated glomerular filtration rate (eGFR) in renal biopsies from IgA nephropathy (IgAN) patients. These findings suggest that TGF-β1 promotes renal fibrosis through the induction of histone H3.3 and HIRA.

## Results

### Histone H3.3 and HIRA expression increase in mouse kidney after UUO

**Figure 1:**
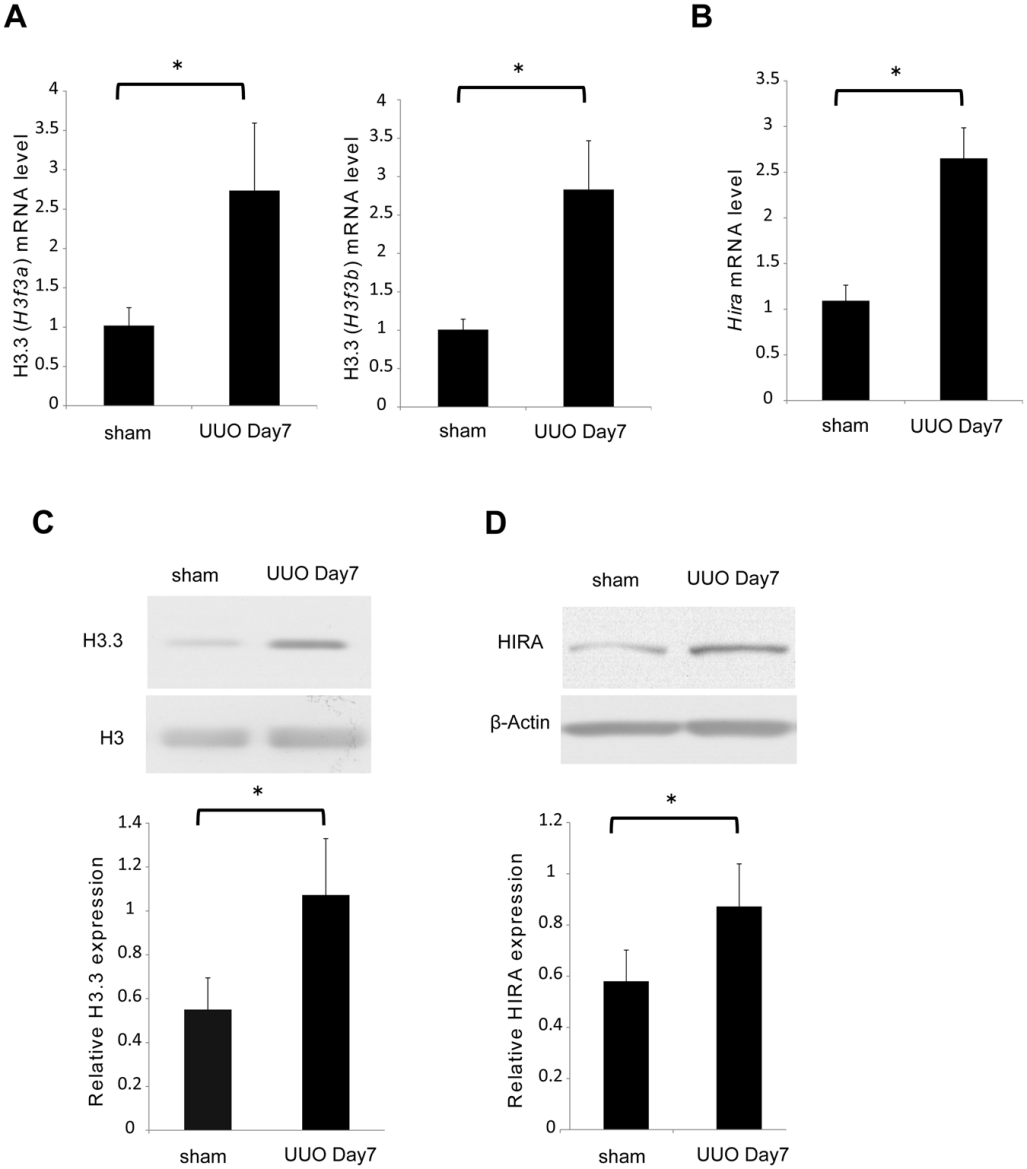
Histone H3.3 and HIRA are up-regulated in the kidney after obstructive injury. (**A**) Histone H3.3 (*H3f3a* and *H3f3b*) and (**B**) *Hira* mRNA levels were determined by quantitative real-time polymerase chain reaction (qRT-PCR) 7 days after sham or UUO surgery. UUO-induced (**C**) histone H3.3 and (**D**) HIRA protein levels were confirmed by western blotting. Histone H3 for histone H3.3 and β-Actin for HIRA were used as internal controls. Data are means ± S.D. *P < 0.05, (*t* test; n = 5 mice per group).

To understand the role of histone H3.3 and HIRA in the progression of tubulointerstitial fibrosis, we first examined the gene expression and protein levels of histone H3.3 and HIRA in UUO mice. As shown in Fig. 1A,B, expression of *H3f3a* and *H3f3b*, encoding histone H3.3, and *Hira* was remarkably increased on day 7 in UUO mice compared with sham controls. Western blot analysis revealed that the protein levels of histone H3.3 and HIRA were increased in UUO mice compared with sham controls, consistent with the increases in mRNA expression (Fig. 1C,D).

### Histone H3.3 and HIRA expression is positively regulated by TGF-β1 in UUO mice and in renal cells

**Figure 2:**
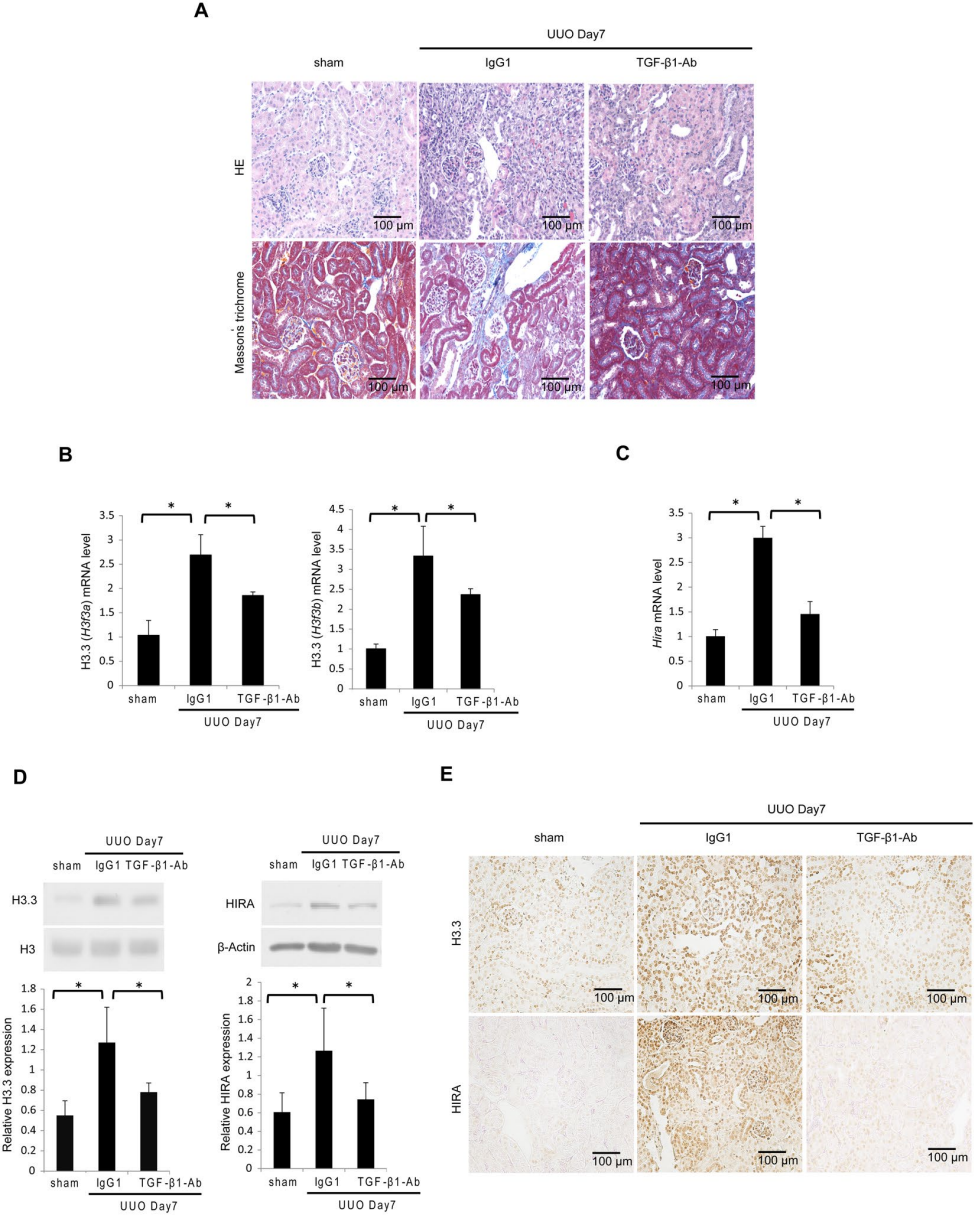
TGF-β1 induces histone H3.3 and HIRA expression and injection of neutralizing TGF-β1 antibody reduces histone H3.3 and HIRA expression after UUO. UUO mice were treated with mouse immunoglobulin G1 (IgG1) or neutralizing TGF-β1 antibody (TGF-β1-Ab). (**A**) Images of HE and Masson’s trichrome staining show histological change in UUO mice with control IgG1 or TGF-β1-Ab. (**B**) mRNA levels of histone H3.3 (*H3f3a* and *H3f3b*) and (**C**) *Hira* were determined in kidneys by qRT-PCR. (**D**) Representative western blot analysis of histone H3.3 and HIRA is shown. Histone H3 for histone H3.3 and β-Actin for HIRA were used as internal controls. Relative levels of expression are shown in the lower panel. (**E**) Images of histone H3.3 and HIRA staining demonstrating the levels of histone H3.3 and HIRA after treatment with control IgG1 or TGF-β1-Ab. Bar = 100 μm. Data are means ± S.D. *P < 0.05, (one-way ANOVA followed by the *post hoc t* test with Bonferroni correction; n = 5 mice per group).

**Figure 3:**
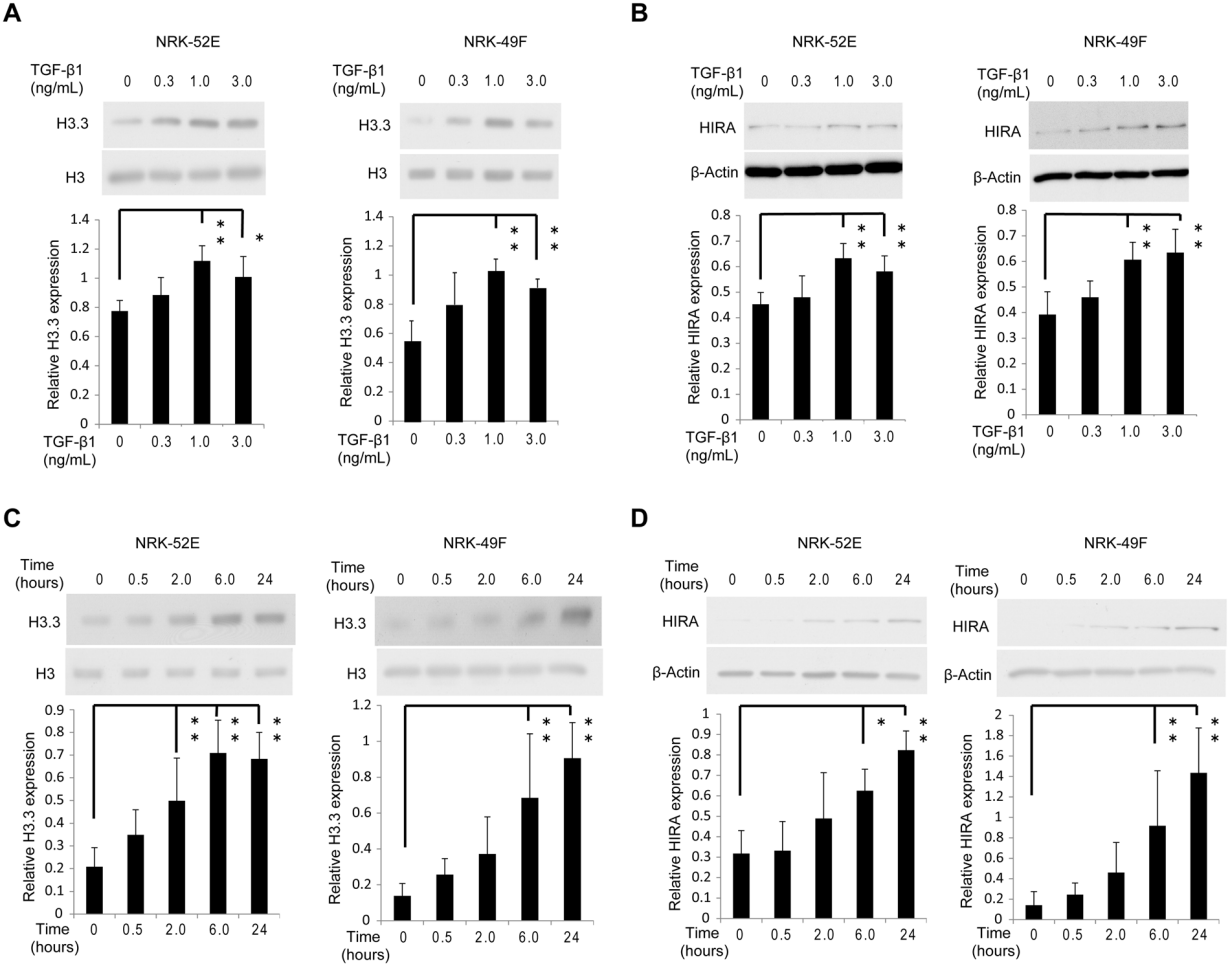
TGF-β1 induces histone H3.3 and HIRA expression in renal epithelial and fibroblast cells. Representative western blotting analysis shows the levels of histone H3.3 and HIRA proteins in TGF-β1-stimulated NRK-52E and NRK-49F cells at various doses (time; 24 hours) for (**A**) histone H3.3 and (**B**) HIRA, and time points (TGF-β1; 1.0 ng/mL) for (**C**) histone H3.3 and (**D**) HIRA. Histone H3 for histone H3.3 and β-Actin for HIRA were used as internal controls. Expression levels were compared with vehicle-treated control. Relative levels of expression are shown in the lower panel. Data are means ± S.D. *P < 0.05, **P < 0.01, (one-way ANOVA followed by Dunnett’s *post hoc* test based on vehicle-treated controls; n = 5 samples per group).

TGF-β1 is a main mediator of renal fibrosis in UUO mice^27^. HE and Masson’s trichrome staining showed histological changes in UUO mice with or without administration of neutralizing TGF-β1 antibody (Fig. 2A). We then examined the regulation of histone H3.3 and HIRA by TGF-β1 *in vivo* and *in vitro*. There was a significant increase in *H3f3a*, *H3f3b* and *Hira* mRNA levels after treatment with mouse IgG1 in UUO mice compared with sham operated mice. Administration of neutralizing TGF-β1 antibody suppressed mRNA levels of *H3f3a*, *H3f3b* and *Hira* in UUO mice^28^ (Fig. 2B,C). Similar results were observed for histone H3.3 and HIRA protein levels (Fig. 2D). Immunohistochemical staining for histone H3.3 and HIRA was predominantly enhanced in the nuclei of tubular epithelial and glomerular cells in UUO mice, and was abrogated by neutralizing TGF-β1 antibody (Fig. 2E). For *in vitro* studies we used normal rat kidney (NRK)−52E cells, a rat kidney tubular epithelial cell line, and NRK-49F cells, a rat kidney interstitial fibroblast cell line. TGF-β1 at doses ≥1.0 ng/mL significantly upregulated histone H3.3 and HIRA protein levels in both cell lines compared with vehicle-treated control (Fig. 3A,B). The same effect was observed 6 hours after stimulation with 1.0 ng/mL TGF-β1 (Fig. 3C,D).

### TGF-β1 induces histone H3.3 and HIRA expression via Smad3 activation

**Figure 4:**
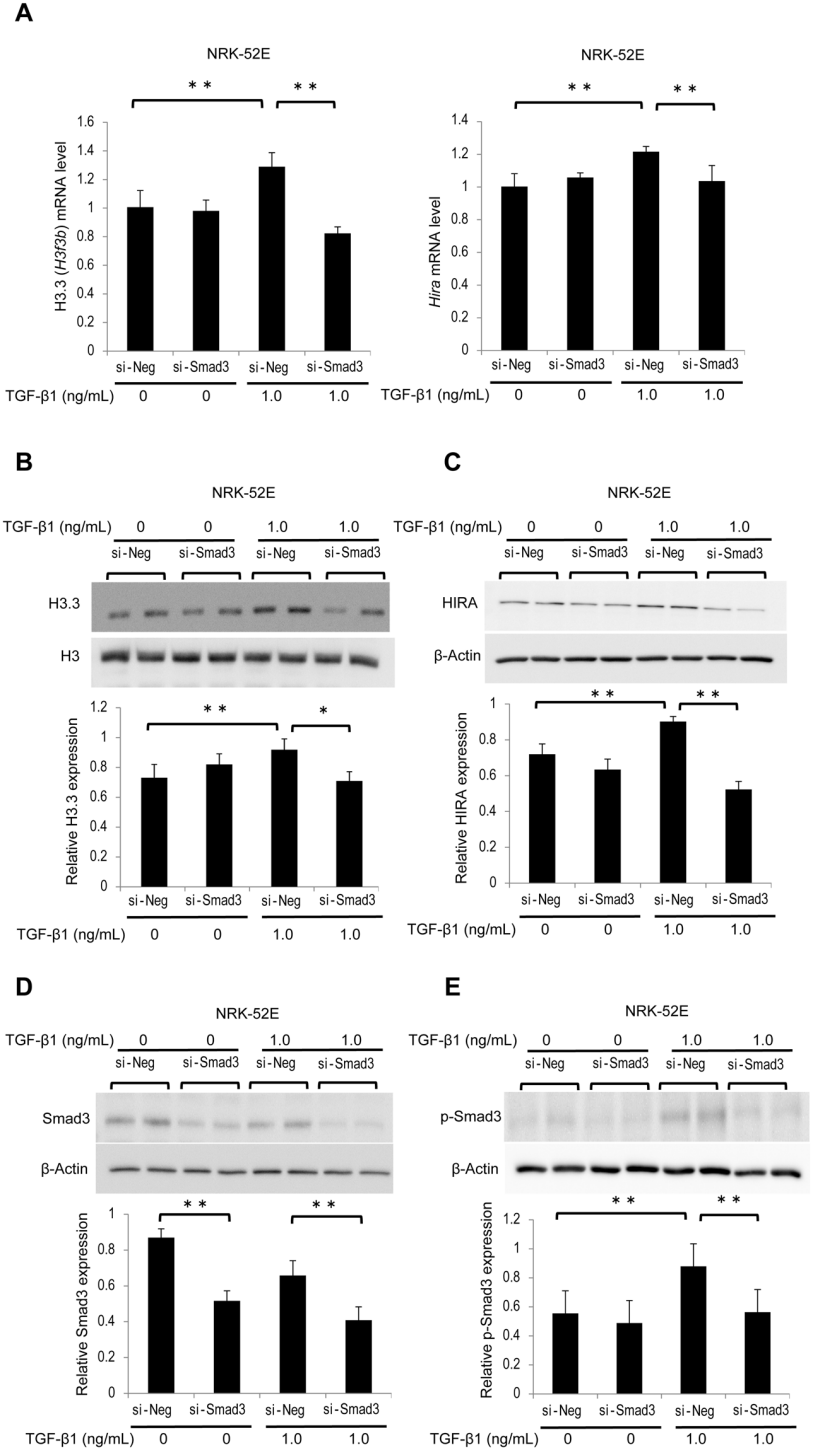
TGF-β1-induced histone H3.3 and HIRA expression is suppressed by knockdown of Smad3 in NRK-52E cells. NRK-52E cells were treated with Smad3 siRNA (si-Smad3) or negative control (si-Neg) oligonucleotides. (**A**) histone H3.3 (*H3f3b*) and *Hira* mRNA levels were determined by qRT-PCR in stimulated NRK-52E cells with or without TGF-β1 (1.0 ng/mL, 24 hours). Representative western blot analysis for (**B**) histone H3.3, (**C**) HIRA, (**D**) Smad3, and (**E**) phosphorylated Smad3 (p-Smad3) in stimulated NRK-52E cells with or without TGF-β1 (1.0 ng/mL, 30 minutes or 24 hours). Because p-Smad3 reaches a peak 30 minutes after TGF-β1 stimulation, this time point was only used in p-Smad3 experiments. Histone H3 for histone H3.3, and β-Actin for HIRA, Smad3, and p-Smad were used as internal controls. Relative levels of expression are shown in the lower panel. Data are means ± S.D. *P < 0.05, **P < 0.01, (one-way ANOVA followed by the *post hoc t* test with Bonferroni correction; n = 5 samples per group).

TGF-β1 is an important mediator during the progression of renal fibrosis through the activation of several signaling pathways, including the Smad3 pathway^29^. We examined whether TGF-β1-induced phosphorylated Smad3 (p-Smad3) is involved in the regulation of histone H3.3 and HIRA expression. NRK-52E cells were treated with small interfering RNA (siRNA) of Smad3 (si-Smad3) or negative control siRNA (si-Neg). After 30 minutes or 24 hours of TGF-β1 stimulation at dose of 1.0 ng/mL, total transfected cells were collected and analyzed. Rat *H3f3a* primers were not commercially available; therefore, we investigated *H3f3b* and *Hira* mRNA levels and protein levels of histone H3.3 and HIRA in NRK-52E cells. *H3f3b* and *Hira* mRNA levels were significantly decreased in NRK-52E cells treated with si-Smad3 (Fig. 4A). Similar results were observed for histone H3.3 and HIRA protein levels (Fig. 4B,C). Likewise, si-Smad3 treatment significantly reduced the levels of Smad3 and TGF-β1-induced p-Smad3 (Fig. 4D,E).

### Knockdown of HIRA attenuates histone H3.3 and TGF-β1-induced fibrogenesis in rat kidney tubular epithelial cells

**Figure 5:**
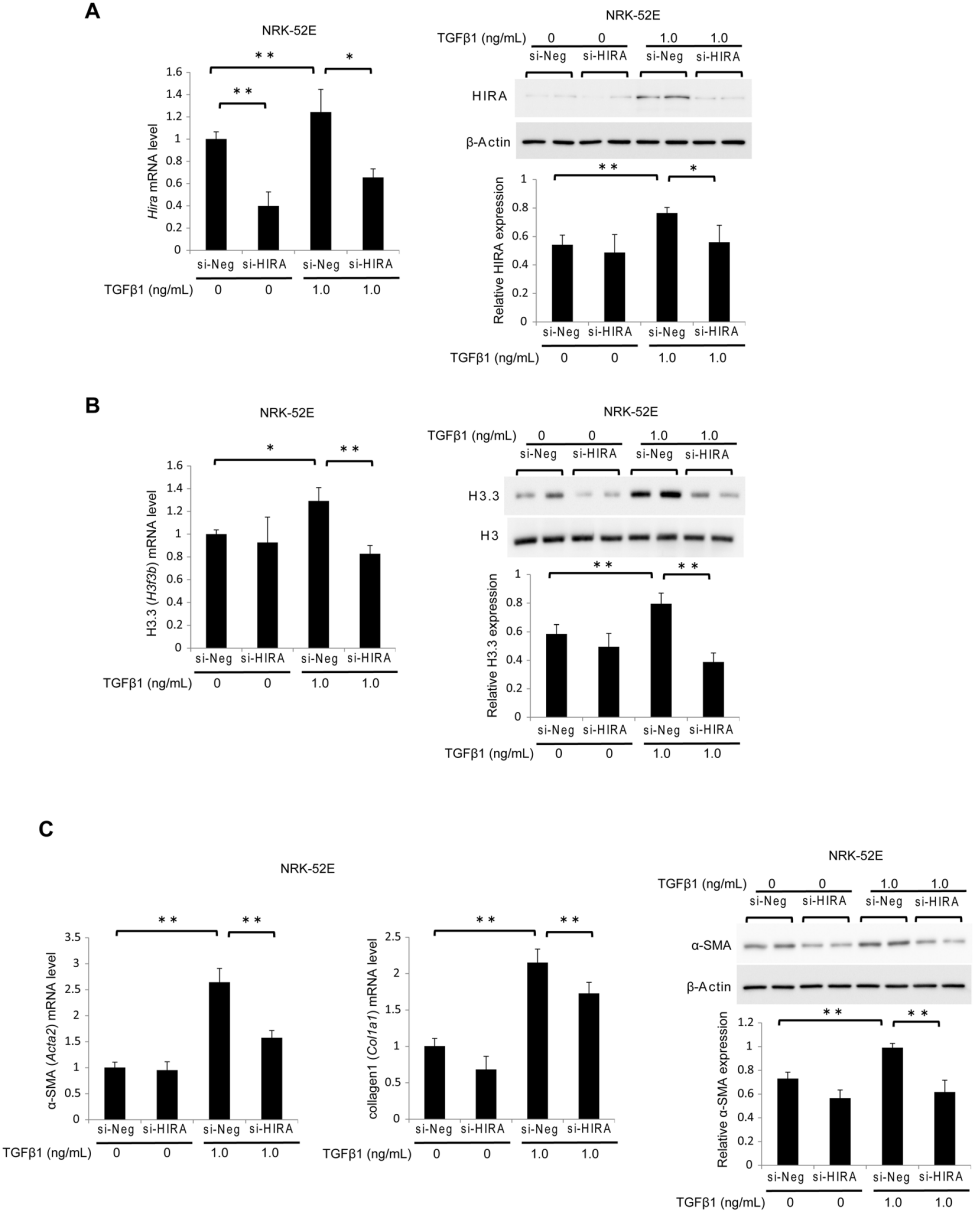
Knockdown of HIRA in NRK-52E cells inhibits TGF-β1-induced fibrogenesis by suppressing histone H3.3 expression. NRK-52E cells were treated with *Hira* siRNA (si-HIRA) or negative control (si-Neg) oligonucleotides. (**A**) *Hira*, (**B**) histone H3.3 (*H3f3b*), (**C**) α-SMA (*Acta2*), and collagen1 (*Colla1*) mRNA levels were determined by qRT-PCR in stimulated NRK-52E cells with or without TGF-β1 (1.0 ng/mL, 24 hours). Representative western blot analysis for (**A**) HIRA, (**B**) histone H3.3 and (**C**) α-SMA. Histone H3 for histone H3.3 and β-Actin for HIRA and α-SMA were used as internal controls. Relative levels of expression are in the lower panel. Data are means ± S.D. *P < 0.05, **P < 0.01, (one-way ANOVA followed by the *post hoc t* test with Bonferroni correction; n = 5 samples per group).

Next, we used siRNA targeting *Hira* (si-HIRA) or negative control siRNA (si-Neg) to investigate whether HIRA promotes fibrogenesis in NRK-52E cells. The effect of si-HIRA in NRK-52E cells was confirmed by reduced HIRA mRNA and protein levels 24 hours after transfection relative to si-Neg with or without 1.0 ng/mL TGF-β1 stimulation (Fig. 5A). Treatment with si-HIRA inhibited TGF-β1-induction of histone H3.3 mRNA and protein levels (Fig. 5B). Levels of α-SMA (*Acta2*) and collagen1 (*Col1a1*) mRNA were very low in the groups without TGF-β1 stimulation, and they progressively increased in the si-Neg and TGF-β1-treated group. In contrast, mRNA levels of *Acta2* and *Col1a1* were significantly decreased in the si-HIRA treated group. In addition, western blotting showed that si-HIRA but not si-Neg attenuated α-SMA protein expression in TGF-β1-treated cells (Fig. 5C).

### TGF-β1 regulates access of fibrosis-related gene promoters to histone H3.3 and HIRA

**Figure 6:**
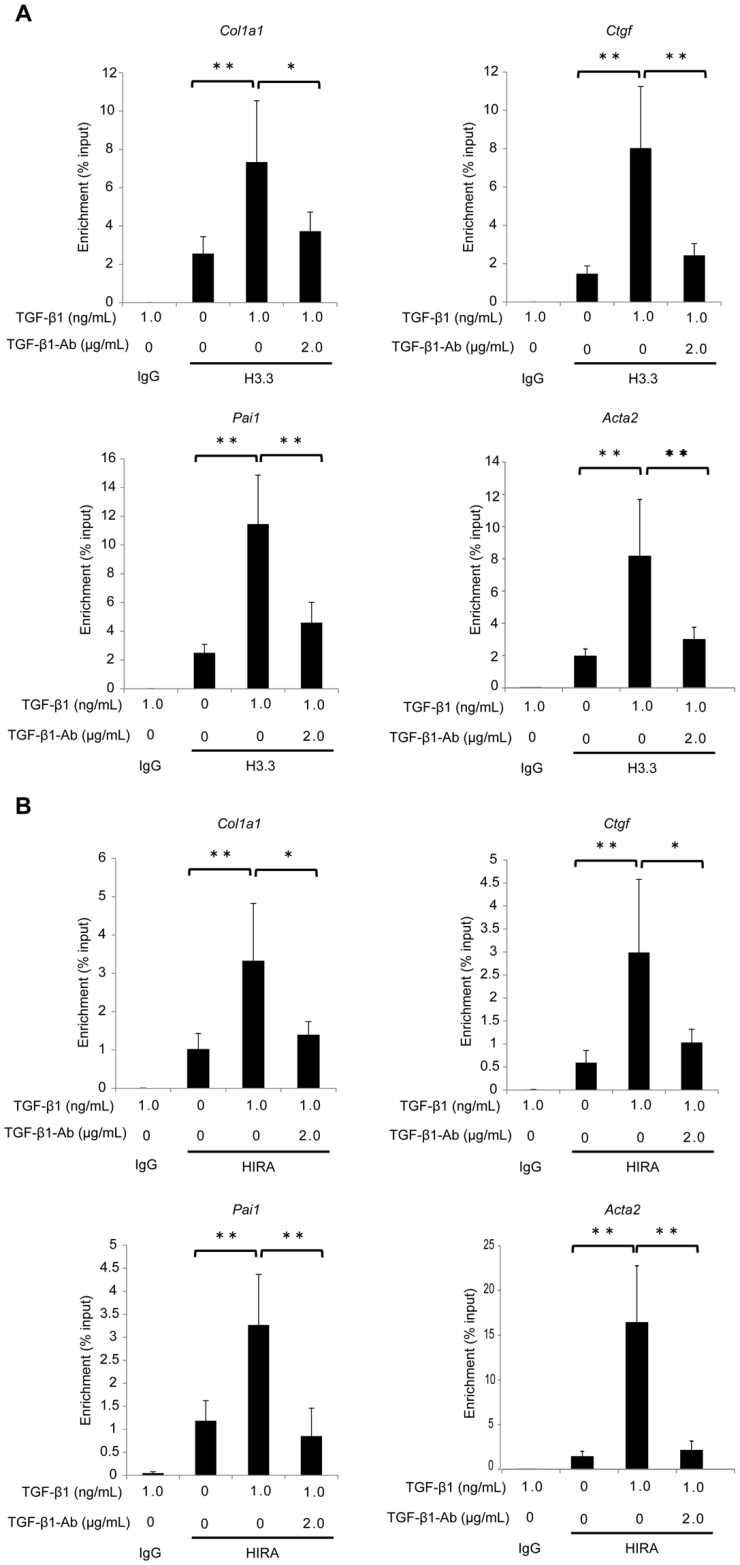
HIRA co-localizes with histone H3.3 at activated promoters of fibrotic genes in NRK-52E cells. Treatment with neutralizing TGF-β1 antibody (2.0 μg/mL) was performed at the same time as TGF-β1 (1.0 ng/mL) stimulation. Chromatin immunoprecipitation (ChIP) analysis of the binding of (**A**) histone H3.3 and (**B**) HIRA proteins to *Col1a1*, *Ctgf*, *Pai1* and *Acta2* promoters in NRK-52E cells is shown. Immunoprecipitated DNA and input DNA were subjected to qPCR. Results were normalized to input DNA and rabbit immunoglobulin G (IgG) was used as a negative control. Data are means ± S.D. *P < 0.05, **P < 0.01, (one-way ANOVA followed by the *post hoc t* test with Bonferroni correction; n = 5 samples per group).

To assess whether TGF-β1 alters the transcriptional activity of H3.3 and HIRA-regulated genes, we conducted ChIP assays in NRK-52E cells. TGF-β1 increased histone H3.3 and HIRA immunoprecipitation of *Col1a1*, connective tissue growth factor (*Ctgf*), plasminogen activator inhibitor-1 (*Pai1*), and *Acta2* gene promoters. Neutralization of TGF-β1 counteracted TGF-β1-induced expression of these genes (Fig. 6).

### HIRA expression correlates with histone H3.3 expression, tubulointerstitial fibrosis and eGFR in kidney biopsies from IgAN patients

**Figure 7:**
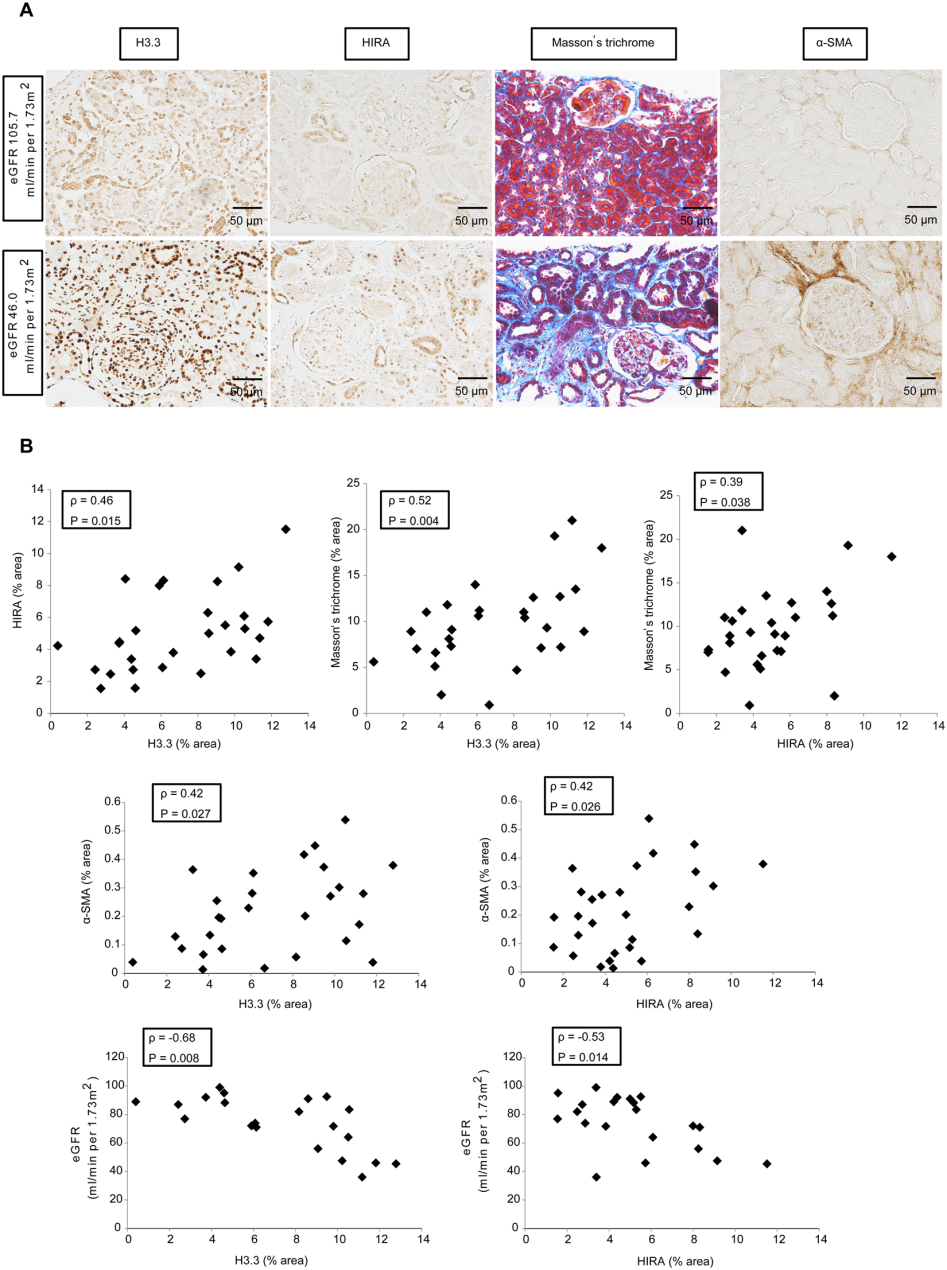
HIRA expression correlates with the expression of histone H3.3, the degree of fibrosis and estimated glomerular filtration rate (eGFR) in human kidney biopsy specimens (n = 28). (**A**) Representative images of histone H3.3, HIRA, Masson’s trichrome staining and α-SMA demonstrate that levels of histone H3.3, HIRA, and the size of fibrotic areas increase with the decline of eGFR in patients with IgA nephropathy (IgAN). Columns show images from the same patient. (**B**) HIRA expression correlated positively with histone H3.3 expression (ρ = 0.46, *P* = 0.015), Masson’s trichrome-positive areas (ρ = 0.39, *P* = 0.038), α-SMA levels (ρ = 0.42, *P* = 0.026) and eGFR (ρ = −0.53, *P* = 0.014). Histone H3.3 expression was positively correlated with Masson’s trichrome-positive areas (ρ = 0.52, *P* = 0.004), α-SMA levels (ρ = 0.42, *P* = 0.027) and eGFR (ρ = −0.68, *P* = 0.008). Spearman’s correlation coefficient test was used. Bar = 50 μm. The Japanese GFR equation based on serum creatinine was used to determine eGFR. eGFR (mL/minute/1.73 m^2^) = 194 × Scr^−1.094^ × Age^−0.287^ × 0.739 (if female).

To examine the clinical significance of histone H3.3 and HIRA in tubulointerstitial fibrosis, we performed immunohistochemistry for histone H3.3 and HIRA on IgAN patient biopsies (n = 28). All patients underwent renal biopsy at Hiroshima University Hospital for the first time between April, 2008 and December, 2010. The average clinical values of the patients were as follows: 36 (31–41) years of age, body mass index was 21 (20–24) kg/m^2^, creatinine clearance was 91 (78–109) mL/minute, eGFR was 75 (59–89) mL/minute/1.73 m^2^, and urinary protein excretion was 0.81 ± 0.78 g/24 hours. We show the details of clinical characteristics related to renal function in Supplemental Table 1. As shown in Fig. 7, immunohistochemistry and histology of biopsies showed that HIRA expression correlated positively with histone H3.3 expression (ρ = 0.46, *P* = 0.015). In terms of fibrosis, Masson’s trichrome-positive area correlated positively with histone H3.3 (ρ = 0.52, *P* = 0.004) and HIRA expression (ρ = 0.39, *P* = 0.038), and α-SMA expression was correlated with histone H3.3 (ρ = 0.42, *P* = 0.027) and HIRA expression (ρ = 0.42, *P* = 0.026). With respect to clinical values, eGFR correlated inversely with histone H3.3 (ρ = −0.68, *P* = 0.008) and HIRA expression (ρ = −0.53, *P* = 0.014), but urinary protein excretion did not show significant correlation with expression of histone H3.3 (ρ = 0.19, *P* = 0.34) or HIRA (ρ = −0.11, *P* = 0.56).

## Discussion

Here, we have shown five new findings. First, expression of histone H3.3 and HIRA increases in a mouse model of renal fibrosis induced by UUO. Second, the TGF-β1-Smad3 pathway regulates the expression of histone H3.3 and HIRA. Third, inhibition of HIRA suppresses TGF-β1-induced fibrogenesis along with a decrease in histone H3.3 expression. Fourth, both HIRA and histone H3.3 immunoprecipitate with promotors of genes involved in fibrosis, such as *Col1a1*, *Ctgf*, *Pai1* and *Acta2*. Finally, histone H3.3 and HIRA expression correlates with not only the degree of fibrosis but also with eGFR in IgAN patient kidneys. This study provides insights into the mechanism by which histone H3.3 and HIRA are involved in TGF-β1-mediated fibrogenesis, and indicates that this histone variant and its chaperone are therapeutic targets for CKD.

Epigenetic regulation of gene expression, for example DNA methylation, histone acetylation and histone methylation, are implicated in renal fibrosis^30–32^. DNA methylation contributes to renal fibrosis, and inhibition of DNA methyltransferase 1 suppresses UUO-induced renal fibrosis^33^, while histone deacetylase inhibitors show an anti-fibrotic effect in mice^34^. Histone methylation also contributes to renal fibrosis through induction of histone methyltransferases, such as SET7/9 and G9a^16,35^. In this study, we demonstrated that expression of histone variant H3.3 and its specific chaperone HIRA were increased in UUO mice. We also showed that levels of histone H3.3 and HIRA in renal biopsies are positively correlated with fibrotic area in IgAN patients. These findings indicate that, in addition to DNA methylation and histone modifications, a histone variant also participates in the progression of renal fibrosis.

We have demonstrated that HIRA plays an essential role in the expression of histone H3.3 in TGF-β1-stimulated NRK-52E cells. An important aspect of histone variant dynamics relates to the mechanism of their incorporation, which involves histone chaperones and can modulate cell fate and the stability of gene expression programs. Histone chaperones regulate the complicated steps folding histones together with DNA to form correctly assembled nucleosomes^36–39^. Replicative histones such as histone H3.1 and H3.2, which are the main components of histone H3, are replicated during S phase in the cell cycle, and are deposited in a DNA synthesis-coupled manner^40^. In contrast, histone H3.3 is incorporated into sites of increased transcriptional activity, independently of both replication and cell cycle^41–43^. We also demonstrated that TGF-β1 increases the transcriptional activity of fibrosis-related genes, and that promoters of these genes are immunoprecipitated with histone H3.3 and HIRA. These findings indicate that histone H3.3 is incorporated into transcriptionally active fibrotic genes by TGF-β1-induced HIRA.

Histone variants function as transcriptional landmarks in response to external stimuli and to maintain networks of gene expression in cooperation with histone modifications^44^. Histone variants are involved in highly-diversified biological processes^45,46^. Among these, histone H3.3 is related to specific cancer types, including pediatric high-grade glioblastoma and certain types of bone tumors^47^. In addition, we confirmed that knockdown of HIRA with siRNA reduced TGF-β1-induced α-SMA expression in rat kidney tubular epithelial cells. Because increased expression of α-SMA-positive myofibroblasts is a hallmark of renal fibrosis^12^, HIRA is considered to participate in the development of renal fibrosis through histone H3.3 induction^48^.

TGF-β1 is a crucial mediator of renal fibrosis progression. In fact, a number of studies show that inhibition of the TGF-β1 signaling pathway ameliorates renal fibrosis^49–51^. At the molecular level, TGF-β1 not only induces transformation of kidney tubular epithelial cells into myofibroblasts but also promotes production of ECM proteins in response to tissue damage^15^. In this study, we showed that UUO-induced histone H3.3 and HIRA expression is suppressed by a TGF-β1-neutralizing antibody, indicating that UUO-mediated TGF-β1 contributes to histone H3.3 and HIRA expression in mice. Moreover, we have demonstrated that phosphorylation of Smad3 is involved with histone H3.3 and HIRA expression. Although p-Smad3 is a transcription factor that upregulates fibrotic genes, TGF-β1-Smad3 signaling also promotes transcriptional activity of these genes^29^.

In this study, si-HIRA did not affect p-Smad3 levels in TGF-β1-stimulated NRK-52E cells (Supplemental Figure S1), suggesting that HIRA is a downstream effector of Smad3. Additionally, we examined mouse kidneys, human kidney biopsies from actual patients, and rat kidney tubular epithelial cells. UUO is as an established model of progressive renal fibrosis^26^. We used UUO mice on day 7 in this study, because severe fibrosis is observed on day 7 after UUO^52^. Another reason is that we cannot observe molecular changes after the development of fibrosis is complete. A number of other studies have investigated renal fibrosis in UUO mice on day 7^53–55^. Moreover, we selected NRK52E cells which are representative and reliable for the investigation of TGF-β1-mediated fibrotic changes^56^. Activation of the TGF-β1-Smad3 pathway is observed during progression of fibrosis in UUO mice and NRK-52E cells, as reported by numerous studies^57–59^. Taken together, our data from UUO mice, rat kidney cells and human biopsy samples indicate that histone H3.3 and HIRA are implicated in TGF-β1-induced renal fibrosis.

Although we showed that inhibition of HIRA expression suppresses TGF-β1-induced α-SMA expression in tubular epithelial cells, the role of HIRA in the progression of renal fibrosis *in vivo* remains unclear. *Hira* knockout mice exhibit embryonic lethality^60^; therefore, further studies with a small molecular inhibitor are needed to assess the potential of HIRA as a therapeutic target for renal fibrosis. In addition, we showed that TGF-β1 induces histone H3.3 and HIRA expression at both mRNA and protein levels, but these changes, although statistically significant, are of relatively small magnitude. However, as shown in Supplemental Figure S2, protein expression is expressed by the formula, target protein/loading control; therefore, a large value for the internal control, rather than a small degree of difference, contributes to the small level in this study. We also showed that neutralizing TGF-β1 antibody and si-Smad3 suppress histone H3.3 and HIRA expression, and that si-HIRA inhibits histone H3.3 and TGF-β1-induced fibrotic change. These findings provide the evidence that TGF-β1 induces histone H3.3 and HIRA expression even though the changes are small in magnitude.

In summary, we showed that histone H3.3 and HIRA expression is increased in UUO mice. We also demonstrated that TGF-β1 upregulates expression of both histone H3.3 and HIRA via a Smad3-dependent pathway. Inhibition of HIRA suppresses not only expression of histone H3.3 but also fibrotic changes in renal epithelial cells. Furthermore, histone H3.3 and HIRA immunoprecipitate with fibrosis-associated promoters. Last, we demonstrated that the expression of histone H3.3 and HIRA is correlated with fibrotic areas and eGFR in patients with IgAN. To the best of our knowledge, no small molecule drug exists that targets HIRA. However, from the data presented here, we suggest that inhibition of HIRA is a promising therapeutic strategy for the treatment of renal fibrosis.

## Concise Methods

### Animals

Eight-week-old male C57BL/6 mice weighting about 25 g were obtained from Charles River Laboratories Japan (Yokohama, Japan). The Institutional Animal Care and Use Committee at Hiroshima University (Hiroshima, Japan) approved all animal experiments (Permit Number: A15-28), and the experiments were performed according to the National Institutes of Health Guidelines on the Use of Laboratory Animals. Mice were randomly assigned to either UUO (n = 5) or sham (n = 5) groups. UUO was performed under general anesthesia (medetomidine, midazolam, and butorphanol) as previously described^61,62^. To investigate the early phase of renal fibrosis progression, mice were sacrificed on day 3 or day 7 after UUO and renal tissues were harvested.

### Mouse treatments

To investigate the expression pathway of histone H3.3 and HIRA in UUO mice, neutralizing anti-TGF-β1 antibody (1D11, 1.5 mg/kg, R&D Systems, Minneapolis, MN) or normal mouse IgG1 (11711, 1.5 mg/kg, R&D Systems) was administered immediately after UUO by intraperitoneal injection. The same treatments were repeated every 48 hours until mice were sacrificed, as previously described^63^. Sham-operated mice were administered same volume of vehicle intraperitoneally.

### Cell Culture and treatments

NRK-52E and NRK-49F cells were purchased from the American Type Culture Collection (Manassas, VA). These cells were maintained in Dulbecco’s modified Eagle’s medium (DMEM) containing 5% fetal bovine serum (FBS) and penicillin/streptomycin. Cells were washed and growth-arrested for 24 hours in DMEM including 0.1% FBS before each stimulation. NRK-52E and NRK-49F cells were stimulated with TGF-β1 at the indicated doses and times. NRK-52E cells were treated with Smad3 siRNA (Invitrogen, Carlsbad, CA) using Lipofectamine 2000 Reagent (Invitrogen) in accordance with the product protocol. After incubation with transfection complexes for 6 hours, the medium was changed, and the cells were stimulated with 1.0 ng/mL TGF-β1 (R&D Systems) for 24 hours. For *Hira* siRNA (Invitrogen), incubation with transfection complexes was for 24 hours. To counteract the effects of TGF-β1 on cultured cells, subconfluent cells were incubated with 2.0 μg/mL anti-TGF-β1 antibody (Abcam, Cambridge, UK) at the same time as TGF-β1 stimulation. Cells were then exposed to TGF-β1 (1.0 ng/mL) with anti-TGF-β1 antibody for 24 hours.

### RNA extraction and quantitative real-time RT-PCR (qPCR)

RNA extraction and qPCR were performed as previously described using an ABI 7500 Fast Real-Time PCR System (Applied Biosystems, Foster City, CA)^64^. Predesigned gene-specific oligonucleotide primers and probes for *Acta2* (assay ID: Mm00725412_s1), *Col1a1* (assay ID: Mm00801666_g1), *H3f3a* (assay ID: Mm01612808_g1), *H3f3b* (assay ID: Mm00787223_s1; assay ID: Rn00821572_g1), and *Hira* (assay ID: Mm00468902_m1; assay ID: Rn01405424_m1) were purchased from TaqMan Gene Expression Assays (Applied Biosystems). The level of Glyceraldehyde 3-phosphate dehydrogenase (*Gapdh*) mRNA was used as an internal control.

### Western blot analysis

Renal tissues or cells seeded in six-well dishes were lysed in 2% SDS sample buffer and sonicated using a Taitec ultrasonic homogenizer VP-050 at 20% power for 30 seconds. Western blot analysis was performed as described previously^65^. Primary antibodies used in this study were as follows: anti-histone H3.3 (Abcam), anti-p-Smad3 (Cell Signaling Technology, Danvers, MA), anti-Smad3 (Cell Signaling Technology), anti-HIRA (Cell Signaling Technology), anti-histone H3 (Cell Signaling Technology), anti-α-SMA antibody (Sigma-Aldrich, St Louis, MO) and anti-β-Actin (Sigma-Aldrich). Horseradish peroxidase-conjugated goat anti-rabbit immunoglobulin G antibody (Dako, Glostrup, Denmark) or goat anti-mouse immunoglobulin G antibody (Dako) were used as Secondary antibodies. Signals were detected by the SuperSignal West Dura or Pico system (Thermo Fisher, Rockford, IL), and the intensity of each band was quantified by ImageJ software (version 1.48p; National Institutes of Health, Bethesda, MD, USA).

### Histology and immunohistochemistry

Histological and immunohistochemical staining of tissue was performed as previously described^66^. Rabbit polyclonal anti-histone H3.3 antibody (Abcam), rabbit polyclonal anti-HIRA antibody (Abcam), and mouse monoclonal anti-α- SMA antibody (Sigma-Aldrich) were used as primary antibodies. Areas of histone H3.3, HIRA, Masson’s trichrome staining or α-SMA were assessed in five random fields (×200 magnification) captured by a digital camera and analyzed using ImageJ. Human kidney specimens were obtained from 28 patients diagnosed with IgAN following renal biopsy at Hiroshima University Hospital between April 2008 and December 2010. The demographic and clinical characteristics of patients are shown in Supplemental Table 1. The research was approved by the Ethics Committee of Hiroshima University (H-2087), and was accordance with relevant guidelines. Informed consent was obtained from each patient.

### ChIP assays

ChIP assays of *Col1a1*, *Ctgf*, *Pai1*, and *Acta2* were performed using a ChIP assay kit (Millipore, Temecula, CA) as previously described^67^. The resulting solutions from NRK-52E cells were incubated overnight at 4 °C with anti-histone H3.3 antibody (Abcam) or anti-HIRA antibody (Abcam). DNA was purified using the QIAquick PCR Purification Kit (Qiagen, Valencia, CA). DNA samples were subjected to qPCR using gene-specific primers as follows:^68^ forward 5′-GGCTGGAGAAAGGTGGGTCT-3′ and reverse 5′-CCCAGGTATGCAGGGTAGGA-3′ were used for *Col1a1*, forward 5′-ATCAGGAAGGGTGCGAAGAG-3′ and reverse 5′-TCCACATTCCTCCGTCTGAA-3′ were used for *Ctgf*, forward 5′-gacaatATGTGCCCTGTGATTGtC-3′ and reverse 5′-AGGCTGCTCTACTGGTCCTTGC-3′ were used for *Pai1* as described previously^32^, and forward 5′-GTAACATAGGCTGGTGAATCCTG-3′ and reverse 5′-GAGTGATCATTTTATGGAAGCCAC-3′ were used for *Acta2* as described previously^69^.

### Statistical analysis

The results are shown as the mean ± S.D. We used the *t* test for comparisons between two groups and one-way analysis of variance (ANOVA) followed by the *post hoc* test using Dunnett’s test or the *t* test with Bonferroni correction for multiple group comparisons. Correlation was calculated using the Spearman correlation coefficient. The presence of a normal distribution was evaluated by the Shapiro-Wilk test. Data with a normal distribution are expressed as the mean ± S.D., and other variables are expressed as the median (25th-75th percentiles). *P* < 0.05 was considered statistically significant.

## Electronic supplementary material

Supplemental Figures
