# Supplementary material for: TGF-β1 promotes expression of fibrosis-related genes through the induction of histone variant H3.3 and histone chaperone HIRA
Source: Sci Rep. 2018 Sep 19;8:14060. doi: 10.1038/s41598-018-32518-8 (PMC6145928; doi:10.1038/s41598-018-32518-8)
Supplement: Supplementary file 1 — Supplemental Figures [file 41598_2018_32518_MOESM1_ESM.docx]

**TGF-β1 promotes expression of fibrosis-related genes through the induction of histone variant H3.3 and histone chaperone HIRA**

Toshihiro Shindo^1^, Shigehiro Doi^1*^, Ayumu Nakashima^1^, Kensuke Sasaki^1^, Koji Arihiro^2^, and Takao Masaki^1*^

^1^Department of Nephrology, Hiroshima University Hospital, Hiroshima, Japan; ^2^Department of Pathology, Hiroshima University Hospital, Hiroshima, Japan

Address Correspondence to:

Shigehiro Doi, M.D., Ph.D. Associate Professor

Department of Nephrology, Hiroshima University Hospital

1-2-3 Kasumi, Minami-Ku, Hiroshima 734-8551, Japan

E-mail: [sdoi@hiroshima-u.ac.jp](mailto:sdoi@hiroshima-u.ac.jp)

TEL: +81-82-257-5960

FAX: +81-82-256-5560

Takao Masaki, M.D., Ph.D. Professor

Department of Nephrology, Hiroshima University Hospital

1-2-3 Kasumi, Minami-Ku, Hiroshima 734-8551, Japan

E-mail: [masakit@hiroshima-u.ac.jp](mailto:masakit@hiroshima-u.ac.jp)

TEL: +81-82-257-1506

FAX: +81-82-257-1508

**Supplemental Data**

**Supplemental Figure S1**

**
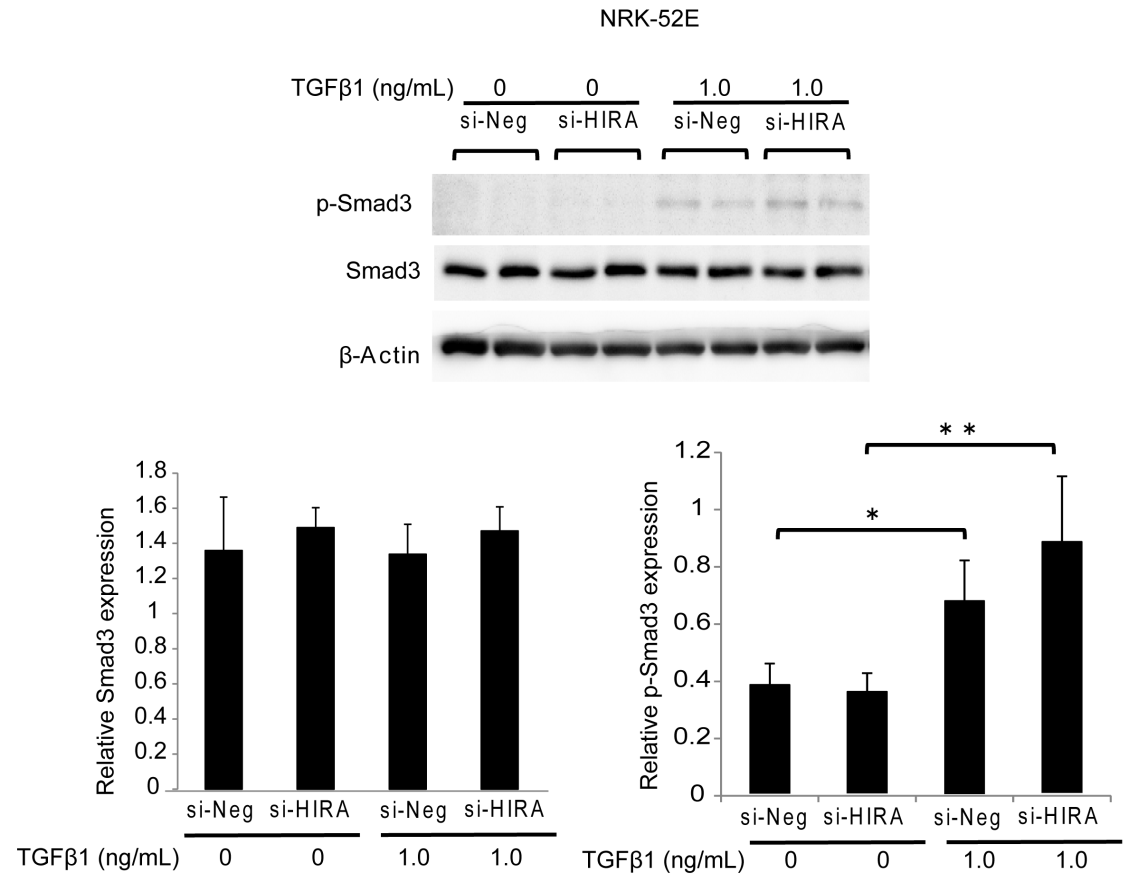
**

**Supplemental Figure S1.** Knockdown of HIRA in NRK-52E cells does not affect TGF-β1-induced p-Smad3 expression. NRK-52E cells were transfected with *Hira* siRNA (si-HIRA) or negative control (si-Neg) oligonucleotides. Representative western blotting analysis shows the levels of Smad3 and p-Smad3 in transfected NRK-52E cells with or without TGF-β1 (1.0 ng/mL, 30 minutes or 24 hours). Total cell lysates were subjected to immunoblotting. Because p-Smad3 reaches a peak 30 minutes after TGF-β1 stimulation, this time point was only used in p-Smad3 experiments. β-Actin for Smad3, Smad3 for p-Smad were used as internal controls. Quantification is shown in the lower panel. Data were analyzed by one-way ANOVA followed by the *post hoc* *t* test with Bonferroni correction. Data are means ± S.D. **P* < 0.05, ***P* < 0.01, n = 5 samples per group.

**Supplemental Figure S2.**


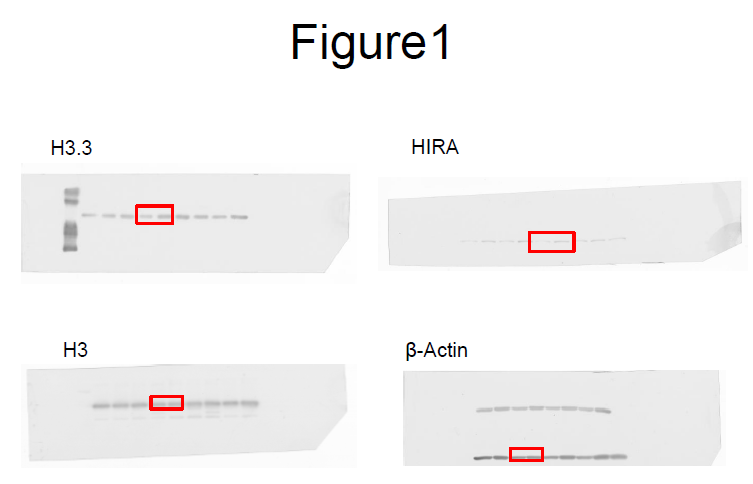


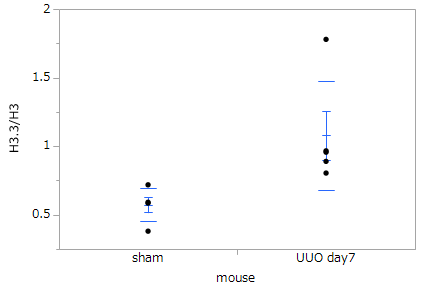

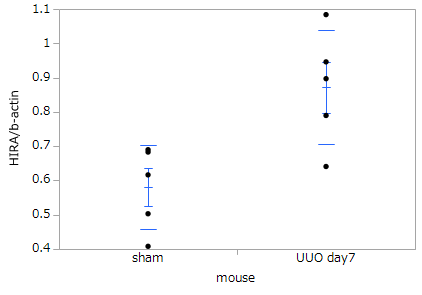


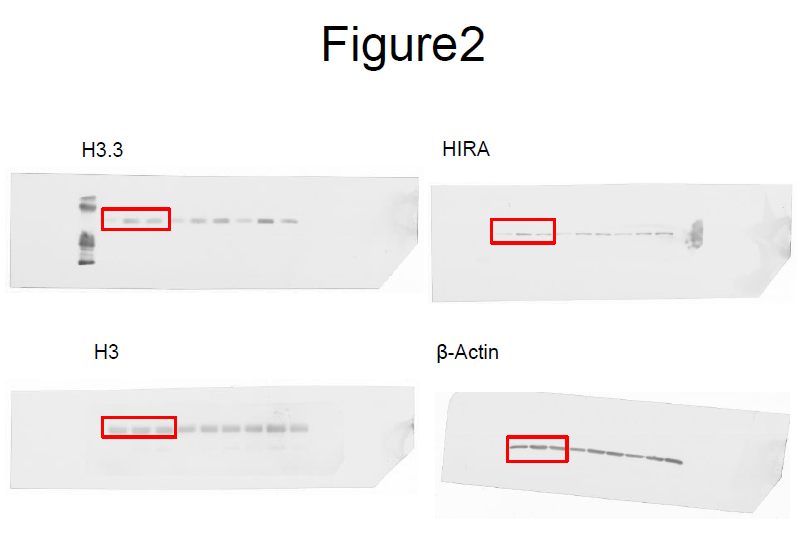


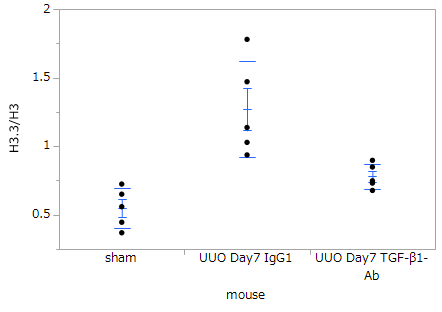


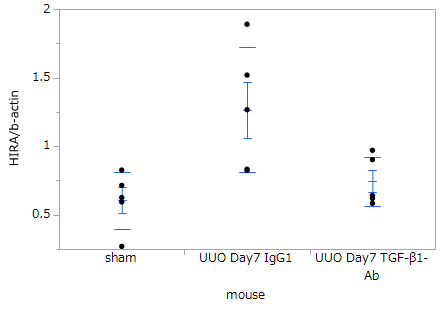


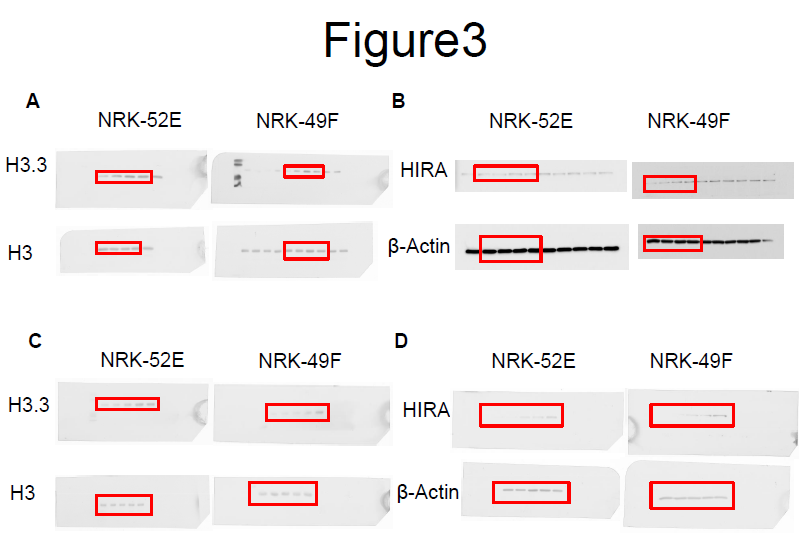


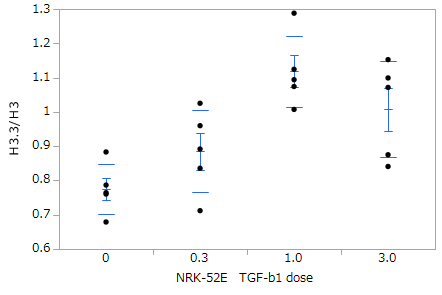

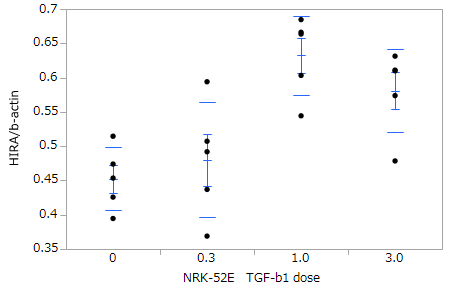

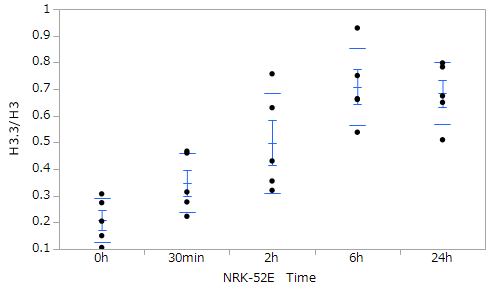

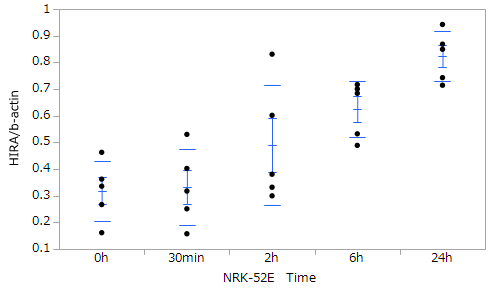

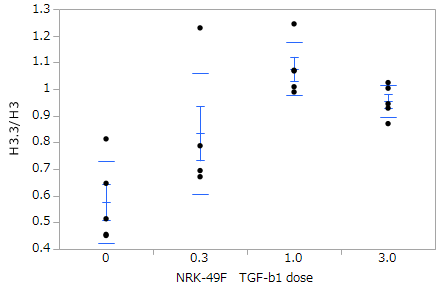

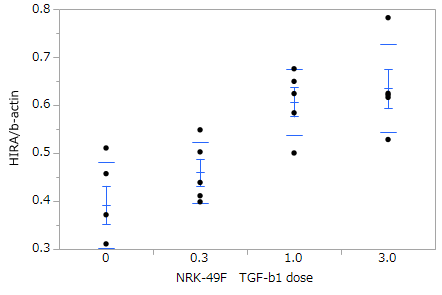

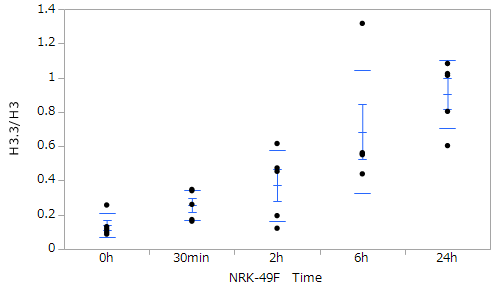

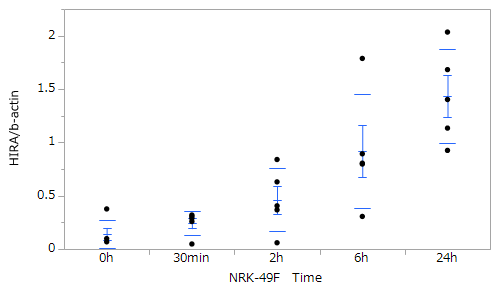


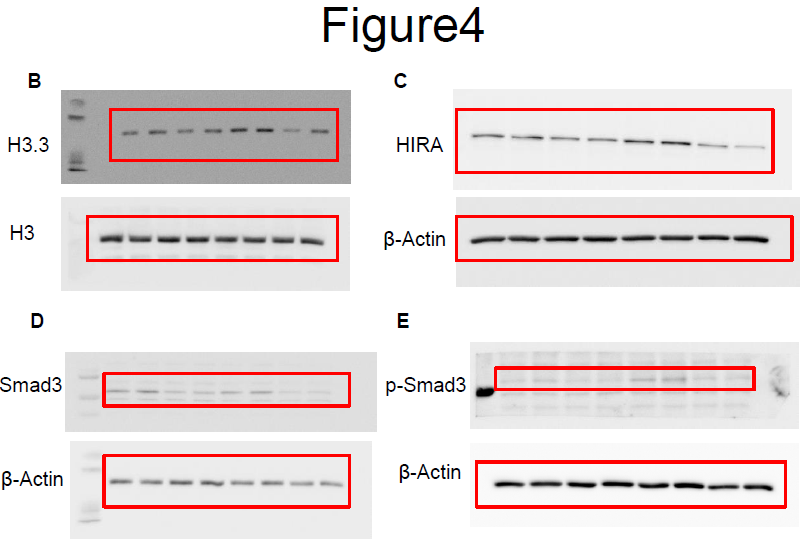


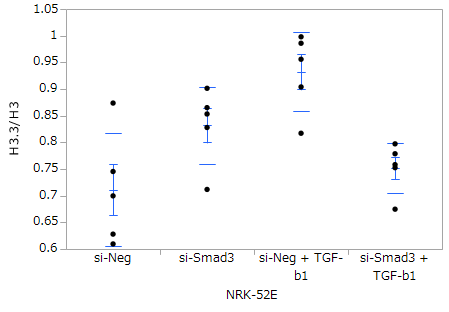

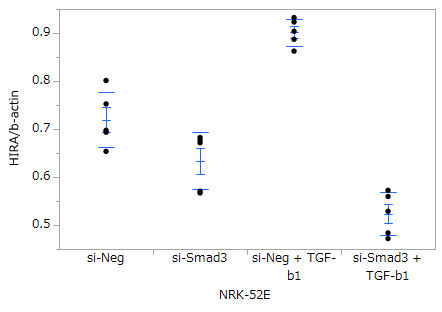

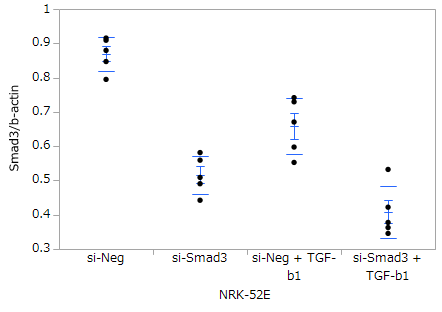

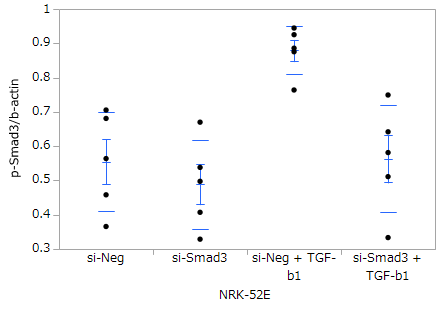


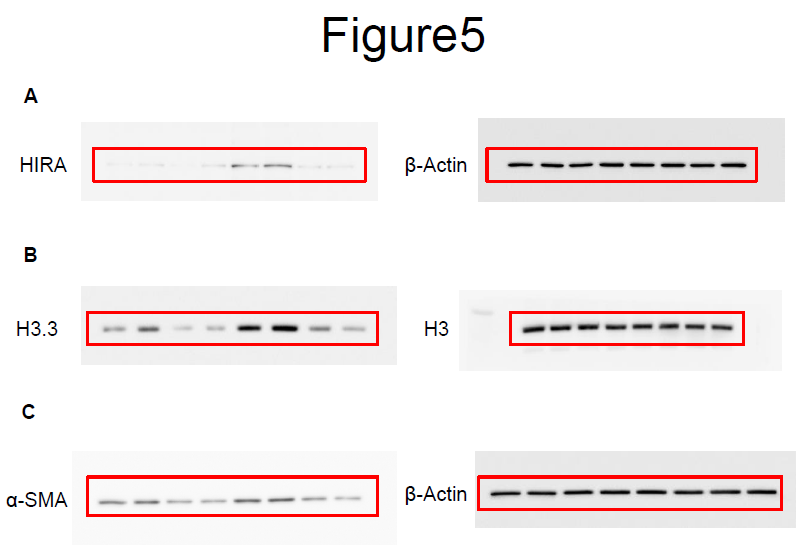


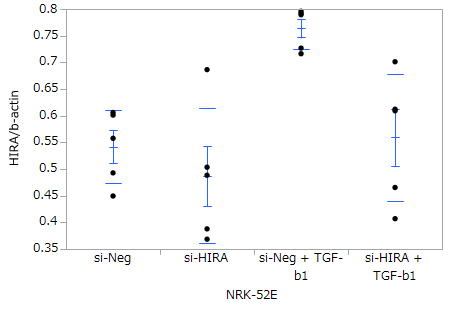

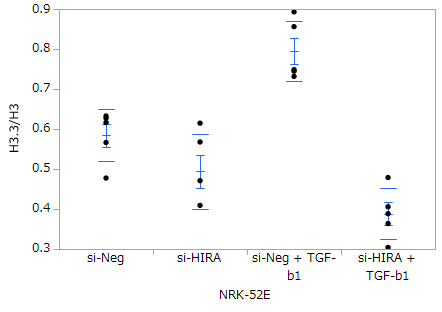

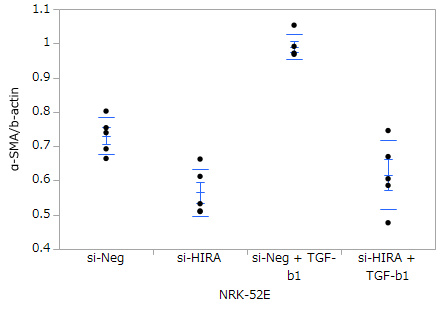


**Supplemental Figure S2.** Full length of Western blots for Figure1-5, and calculate intensities for all the blots. The red boxes indicate the cropped regions.

**Supplemental Table 1. Clinical characteristics related to renal function of IgAN patients**

| Variables | Total (n = 28) |
| --- | --- |
| Age | 36 (31-41) |
| Male sex | 55% |
| Body mass index | 21 (20-24) |
| Renin-angiotensin system inhibitor | 14% |
| Systolic blood pressure (mmHg) | 113 ± 14 |
| Diastolic blood pressure (mmHg) | 68 ± 10 |
| Total urinary protein (g/day) | 0.81 ± 0.78 |
| S-Cre (mg/dL) | 0.89 (0.73-1.04) |
| eGFR (ml/min) | 75 (59-89) |
| Creatinine clearance (ml/min) | 91 (78-109) |
| Total cholesterol (mg/dL) | 192 (167-248) |
| Triglyceride (mg/dL) | 123 ± 89 |
| LDL cholesterol (mg/dL) | 117 ± 39 |
| HDL cholesterol (mg/dL) | 68 (50-81) |
| Oxford classification |  |
| Mesangial hypercellularity | M1: 79% |
| Segmental glomerulosclerosis | S1: 61% |
| Endocapillary hypercellularity | E1: 4% |
| Tubular atrophy / interstitial fibrosis | T1: 39%, T2: 7% |

Continuous variables are expressed as the mean ± SD or median (interquartile range) and categorical variables by percentage.

eGFR: estimated glomerular filtration rate

The Japanese GFR equation based on serum creatinine was used to calculate eGFR.

eGFR (ml/min/1.73 m^2^) = 194 × Scr ^− 1.094^ × Age ^− 0.287^ × 0.739 (if female).

S-Cre: Serum creatinine

LDL: low-density lipoprotein, HDL: high-density lipoprotein
